# Boosting New Electrochemical Reactor Designs to Improve the Performance in H_2_O_2_ Production Using Gas Diffusion Electrodes

**DOI:** 10.1021/acssuschemeng.4c08826

**Published:** 2025-02-20

**Authors:** Taynara Oliveira Silva, Rafael Granados-Fernández, Justo Lobato, Marcos R. V. Lanza, Manuel Andrés Rodrigo

**Affiliations:** † São Carlos Institute of Chemistry, 28133 University of São Paulo (USP), 400, São Carlos, São Paulo 13566-590, Brazil; ‡ Department of Chemical Engineering, 16733 Universidad de Castilla-La Mancha, Ciudad Real 13071, Spain

**Keywords:** mechanical design, cell performance, comparison cells, fluid dynamics, electrosynthesis, hydrogen peroxide, gas diffusion electrode, scale-up

## Abstract

This work presents a novel electrochemical cell design, developed using 3D printing technology, which enhances turbulence within the cell to promote increased hydrogen peroxide production. This new design is compared to a conventional flow cell that utilizes the same electrodes, membrane, and interelectrode distance, which has demonstrated strong performance in previous studies. Fluid dynamics and H_2_O_2_ production are analyzed in both reactors to assess their performance. Additionally, a scale factor of 12.5 is applied to the new concept to evaluate its effectiveness on a larger scale and increase the technology readiness level (TRL). The results demonstrate Faradaic efficiencies of 90% and energy consumption as low as 13 kW h kg^–1^, placing them among the highest reported in the literature. The use of identical materials and operating conditions underscores the critical role of mechanical design in electrochemical cells, suggesting that future research in environmental electrochemical technology should prioritize cell designs tailored to specific target processes.

## Graphical abstract

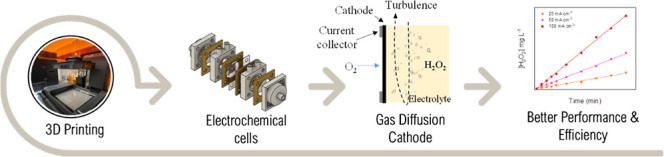

## 1 Introduction

In recent years, the use of 3D printing technology has been widely extended across many research groups because of the importance of the improvements that can be obtained with the direct manufacturing by researchers of prototypes adapted to their necessities.^1–16^ This topic is of particular interest in electrochemical engineering as the performance of electrochemical cells is heavily influenced by geometric factors. Since these cells function as heterogeneous reactors, efficient mass transfer plays a critical role in determining overall effectiveness.^17,18^ This was anticipated in a prospective review made a decade ago by the Working Party on Electrochemical Engineering of the European Federation of Chemical Engineering,^19^ but current outcomes have been even better than expected. The efficient transport of reagents to the electrode surface, or the efficient transport of products electrogenerated toward the bulk of the electrolyte, often controls the rate and selectivity of electrochemical processes. This aspect of mass transport can have a more significant impact on performance than optimizing other factors, such as electrode materials or operating conditions.^20,21^

Effects of the mechanical design of cells have been typically evaluated using computational fluid dynamics (CFD) modeling.^18,22–25^ While mathematical modeling is a powerful tool, its complexity can be quite high, and selecting the right model parameters is often crucial for accurately interpreting the results. Additionally, the mathematical complexity and occasional lack of robustness in the predictions can limit the usefulness of the simulations. In many cases, the reliability of results from real experimentation with physical cell prototypes, or iconic simulation tools, can surpass that of purely mathematical simulations.^21,26,27^ This fact is even more tricky when the occurrence of biphasic flows is noticed. Those flows are characteristic of electrochemical cells operating with aqueous electrolytes, in which the oxidation and reduction of water to form, respectively, oxygen and hydrogen are very important and also in processes in which gases are flowed into the electrolyte, such as those associated with (1) electrochemically assisted reactive-absorption treatment used to remove VOCs or odorous substances from gaseous streams,^28,29^ (2) the electrochemically assisted fixation of carbon dioxide,^30^ or (3) the production of hydrogen peroxide (H_2_O_2_) by cathodic reduction of oxygen.^31–35^

This latter case is of paramount importance. H_2_O_2_ is typically obtained by the anthraquinone process,^36^ which, despite its economic advantages concerning the state of the art of the electrochemical processes, exhibits serious environmental problems that may be easily avoided with the cathodic reduction processes. In recent years, a huge effort has been made by many researchers to develop new concepts of pressurized cells to increase the solubility of the raw matter (oxygen) or to develop efficient gas diffusion cathodes (GDCs) to operate in atmospheric-pressure electrochemical cells most effectively. Very high efficiencies, exceeding 80%, are currently being achieved. However, it has been suggested that further improvements in electrode manufacturing can result only in marginal gains. A more significant breakthrough could come from optimizing cell design, specifically by enhancing turbulence and improving gas release. These design improvements could potentially bring efficiencies closer to the theoretical maximum of 100%.^37–44^

There is a lack of studies in which the role of the electrochemical cell is evaluated and, as well, in which the effect of the scale-up is tackled. Both shortcomings are going to be studied in this work by comparing a very effective flow cell that has demonstrated a highly efficient performance in previous works^45–49^ with a tailored cell that it is, in fact, an adaptation of a cell, previously developed for the highly efficient production of ozone,^50^ to the cathodic production of H_2_O_2_ with the implementation of a gas diffusion electrode (GDE) and a simple cationic exchange membrane (CEM).

## 2 Materials and Methods

### 2.1 Manufacture of Electrochemical Cells with 3D Printing

Prototypes were designed using Fusion 360, from Autodesk, with an educational license, and printed using a Form3 stereolithography machine from the Formlabs company. A transparent resin was used as a material for printing (Clear V4). Subsequently, laminator software was used to generate the printing files. To enhance the material properties even more, the components underwent a 10 min immersion in isopropyl alcohol followed by curing under UV-light and a temperature of 60 °C for 15 min. All cells consist of four pieces integrated mechanically with seal gaskets and screws to the assembly.

### 2.2 Experimental Setup

In both cases, the same electrode size and flow rate are used. In addition, the novel design is going to be evaluated at two different electrode sizes with a scale-up factor of 12.5 to check whether there are any important effects associated with the size of the electrochemical cell. Figure 1 shows the sketch of the mechanical design of the two types of electrochemical cells aimed to be compared in this work, in which the expected flow patterns are highlighted.

**Figure 1:**
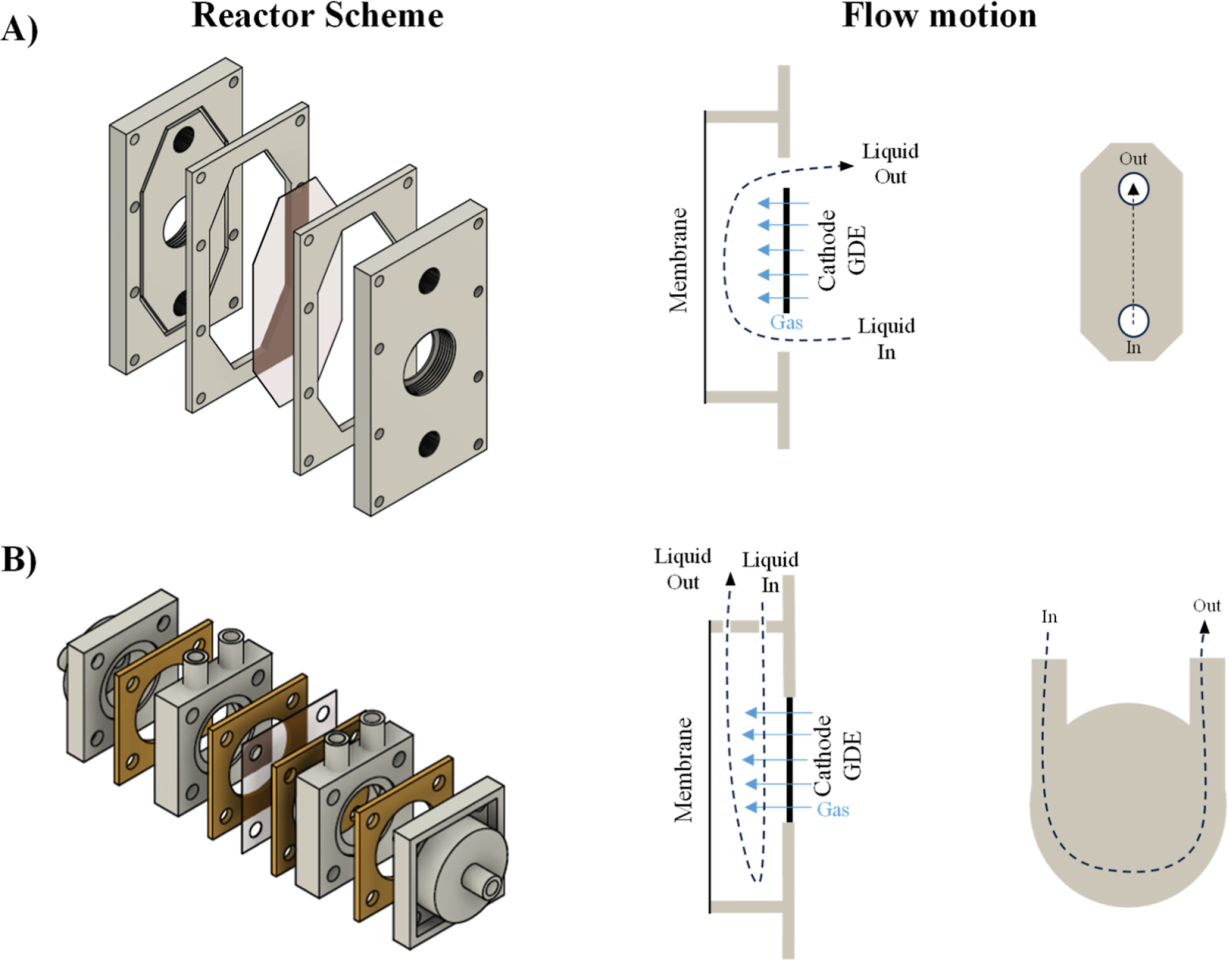
Sketch of the cell concepts aimed to be compared in this work with indications of the main electrolyte and gases flows expected.

Thus, Prototype 1 (Figure 1A) is a conventional flow cell built in a mechanical workshop with a primary electrolyte flow pattern parallel to the electrodes. Prototype 2 (Figure 1B) is designed to produce a highly turbulent centrifugal flow pattern inside the cell, and that was successfully tested with a proton exchange membrane (PEM) configuration to produce ozone. This design is adapted in this work to host a GDE as a cathode keeping the same electrolyte flow pattern. For a fair comparison, both cells are divided by the same type of PEM, and both prototypes are equipped with the same GDE (size: 20 cm^2^, circular shape, and thickness: 1.0 mm) and plate anodes (size: 20 cm^2^, circular shape, and thickness: 1.0 mm). Further details about the electrodes can be found elsewhere.^47^ As schematized in Figure 1, key differences are found in the inlet and outlet of the electrolyte (because the same flow rates were kept in the comparison made in this work) and in the shape of the inner gap. Additionally, to go further in the knowledge about the influence of the electrolyte flow patterns, Prototype 2 was designed and manufactured in two different sizes capable of hosting electrodes of 20 and 1.6 cm^2^ (scale-up factor 12.5). In both cases, the interelectrode gap was kept at the same value (24 mm).

The three cells were equipped with the same types of anodes and cathodes. As anodes, dimensionally stable anode (DSA-Cl_2_) electrodes were purchased from the DeNora S.P.A., Brazil. As cathode, GDE manufactured by hot pressing (2 tons for 25 min at 210 °C of temperature) according to the procedures described^51^ were made using carbon black Printex L6—CPL6 (Evonik Brazil ltd.), PX30 carbon cloth from Zoltek, and poly­(tetrafluoroethylene) (PTFE) from Sealflon. Electrodes were connected to a power supply ES030–10 (15 V/10 A) from Delta Elektronica. Production of H_2_O_2_ was made under galvanostatic conditions, and current densities were modified in the different tests of 25, 50, and 100 mA cm^–2^. Both compartments of the cell were hydraulically connected to reservoir tanks (with a capacity of 2.0 L) that contained 1.0 L of each electrolyte (Na_2_SO_4_ = 0.5 mol L^–1^) with pH 2.5, adjusted with H_2_SO_4_. The electrolytes were recirculated between the reservoir tanks and the electronic compartments by two peristaltic pumps (Percom N-M II from the JP Selecta group) with a flow rate of 250 mL min^–1^. The temperature of the electrolytes was kept with a thermostatic bath fixed at 25 °C (Tectron-200 27 from JP Selecta group). The flow rate of the O_2_ fed to the GDE was 50.0 mL min ^–1^.

Figure 2 shows a schematic diagram of the two prototypes used to produce H_2_O_2_. In the case of (A), four rectangle pieces are assembled with the membrane, and a circular cover for electrodes, cathode, and anode appears in the middle of the reactor. This circular cover is where the current collector is located. The flow is linear from the bottom area to the upper zone, where the liquid exit is by overflow. For this electrochemical reactor, the distance between the electrodes is 24 mm with a total volume of 300 mL in a rhomboid form, which is the major diagonal in the *Z* direction, while prototype B is composed of four-square pieces, and in the middle is located the membrane. In this prototype, the volume for the electrolyte is circular, with 42 mL of internal total volume. This circular shape inside the reactor favors turbulence at the inlet and outlet in the liquid and gas phases.

**Figure 2:**
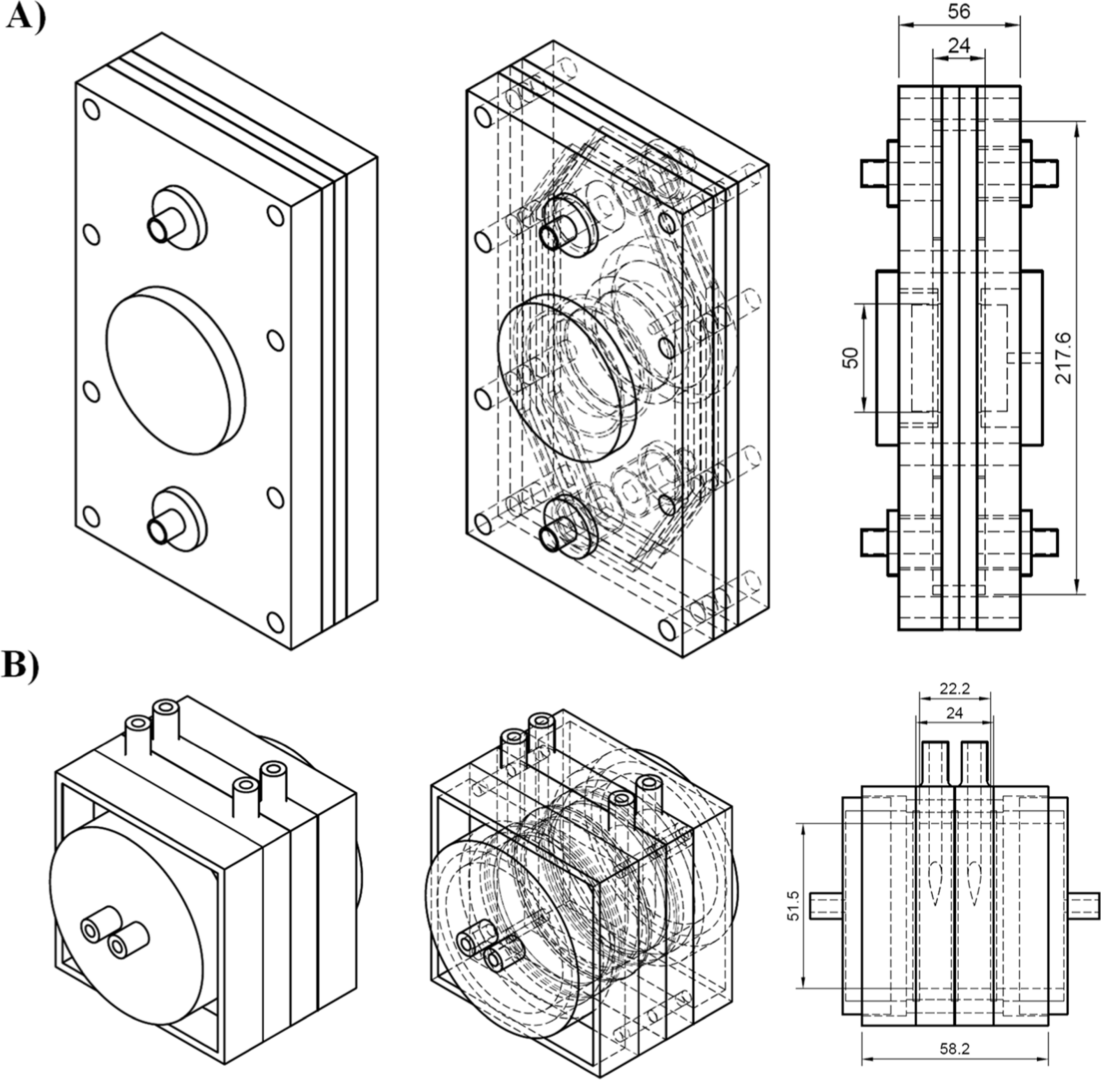
Schematic diagram of the different prototypes, geometry, and dimensions (mm). (A) Prototype 1 and (B) Prototype 2.

### 2.3 Analytical Procedures

H_2_O_2_ in solution was quantified using UV–vis spectroscopy (Shimadzu UV-1700 spectrophotometer) by colorimetry with titanium­(IV) oxysulfate solution (at λ = 408 nm).

## 3 Results and Discussion

### 3.1 Fluid Dynamic Characterization

Before testing the electrochemical performance of the two cells compared in this work, it was considered important to work on the flow pattern characterization as the same electrochemical parameters are going to be applied to both cells. As shown in Figure 1, Prototype 1 corresponds to the most reported type of electrochemical flow cell equipped with a GDE found in the literature. This cell has been successfully used in previous works to produce H_2_O_2_.^45,47,52^ Gas is introduced into the cathode and forms the reaction product on the three phase boundaries (tpb) of its surface. Meanwhile, surplus gas and products are efficiently dragged by the electrolyte with a bottom-top perpendicular flow that prevents the accumulation of gases in the interelectrode gap, favoring their release. The shape of the interelectrode compartment is designed to attain a uniform distribution of electrolyte, preventing the formation of important nonuniform current distributions in the electrodes and also prevents the formation of hydraulic short circuits in the electrolyte due to its sufficient salt concentration, which ensures conductivity within the system (cathode, electrolyte, and anode). In contrast, Prototype 2 was an adaptation to host GDE of a previously designed cell of our group.^50^ In this case, the electrodes were fitted to the external frames of the cell, and one of them was designed to distribute air through a cavity that allows to obtain a suitable backpressure. The fluid dynamic characterization of that cell is reported elsewhere,^53^ and it points out the promotion of turbulence in the cell by centrifugal flow patterns, which differs importantly from the linear flow patterns that are typically developed in flow cells.

To know more about the difference in the electrolyte flow patterns in both types of cells, it was decided to simulate both using CFD tools. Figure 3 compares the expected fluid velocity profiles in the cathodic chamber of the electrochemical cells evaluated in this work considering the flow rate used in the test carried out (250 mL min^–1^) and the geometric characteristics of each cell. In the case of Prototype 2, the two sizes that are compared for scale-up purposes were simulated. Autodesk CFD software was used for the simulation of CFD. For the modeling, a laminar regime was selected, and an adaptive mesh setting with three cycles of 150 iterations each was employed until mesh independence was achieved with over 97% of both pressure and velocity magnitudes. The boundary conditions included a uniform velocity at inlets (with the fluid velocity determined by the flow rate of 250 mL min^–1^), calculated from the internal diameter of the inlet piping and a gauge pressure of 0 bar at the outlet. For Prototype 1, Prototype 2 (large electrode), and Prototype 2 (small electrode) simulations analyzed a mesh with 491259, 254433, and 676632 elements, respectively. After completing the calculations, plane and isosurface plots were generated to compare the fluid’s linear speed along the different reactors. A color-code is used to identify different speeds in mm s^–1^.

**Figure 3:**
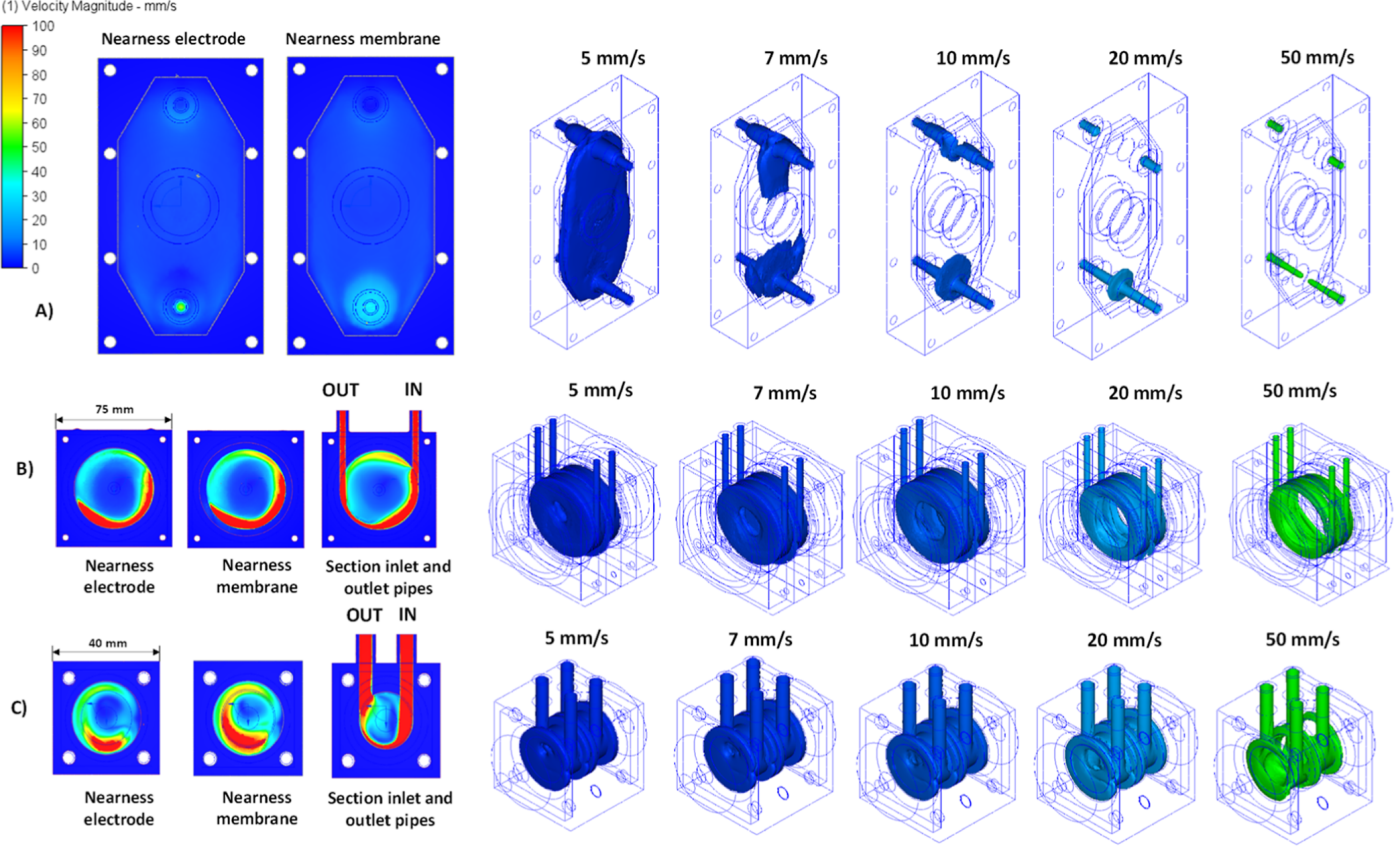
Comparison of the velocity profile isosurfaces: (A) Prototype 1 (electrode size 20 cm^2^). (B) Prototype 2 (electrode size 20 cm^2^). (C) Prototype 2 (electrode size 1.6 cm^2^).

More specifically, in Prototype 1, the inlet flow is perpendicular to the internal volume of the reactor. This flow collides with the membrane, generating a higher velocity where radial distribution occurs (velocity of 30 mm s^–1^) of the fluid to the rest of the cell. However, as the fluid ascends, it loses velocity since there is no element inside the reactor to create turbulence. On the other hand, in Prototype 2, the inlet and outlet flow is tangential to the internal cavity of the reactor, causing a centrifugal flow inside (swirl), which increases the velocity within the system.

As seen, for the same liquid flow rate fed (250 mL min^–1^), that is, for the same power applied for pumping, Prototype 2 attains a more vigorous profile of velocities, and hence, it is expected that it reaches a higher mass transfer coefficient and a more efficient release of gases. In the isosurface curves, while Prototype 1 has an average fluid velocity below 5 mm s^–1^, the cell compartment of Prototype 2, regardless of the size of the cell, is above 50 mm s^–1^. In addition, opposite to Prototype 1, in which the uniformity of the blue color indicates a uniform velocity of the fluid in all parts of the electrolyte compartment (as it is the design purpose targeted in this type of cells), in the case of Prototype 2, red zones indicate a nonuniform distribution that may help gas release as the gas is added in the central position. At this point, it is important to consider that the gap is the same in the two prototypes, but the volume is different because of the large zones required in Prototype 1 for producing the uniform flow distribution.

On the other hand, considering the difficulties and the huge uncertainness associated with CFD modeling of biphasic flow, whose results are known to depend strongly on model parameters proposed, to have a more realistic view, effects of the gas dosing on the two cell prototypes were evaluated experimentally by measuring mass transfer coefficients in the cell under three different dosing rates of gas. Figure 4 shows mass transport coefficients calculated with the ferro-ferricyanide method in these three different cases: feeding the GDE with air at the same flow rate used in the test of production of H_2_O_2_, which is going to be described in the next section (50 mL min^–1^), doubling it (up to 100 mL min^–1^), and in the absence of gas flow rate (0 mL min^–1^). This type of standardized test^54^ finds the limit current intensity *I*_lim_ by carrying out a *I*/*V* polarization curve looking for a plateau in the intensity (Figure S1. Supporting Information) that is interpreted in terms of a control of the process by the transport of ferrocyanide reagent to the electrode surface. For this purpose, an electrolyte solution was prepared with 0.50 M Na_2_CO_3_ as the supporting electrolyte, 0.05 M K_4_Fe­(CN)_6_ (the compound to be anodically oxidized), and 0.10 M K_3_Fe­(CN)_6_ (the compound to be cathodically reduced). The concentration of ferricyanide was twice that of ferrocyanide, to ensure that the oxidation of ferrocyanide was limited at the anode. The ion flux to the anode results from a concentration gradient and convection due to fluid flow. Velocity at the anode is zero, and the potential gradient is negligible due to the presence of the supporting electrolyte. In these conditions, ion flux to the anode is primarily governed by diffusion, and the mass transfer coefficient on the anode surface can be related to the limiting current. Then, the mass transfer coefficient is calculated according to eq 1, where *C*_bulk_ is the concentration of K_4_Fe­(CN)_6_ and A is the geometric electrode area, F is the Faraday constant, and *n* is the number of electrons exchanged.

1 $$K_{m}=\frac{I_{\lim}}{nFC_{bulk}A}$$

**Figure 4:**
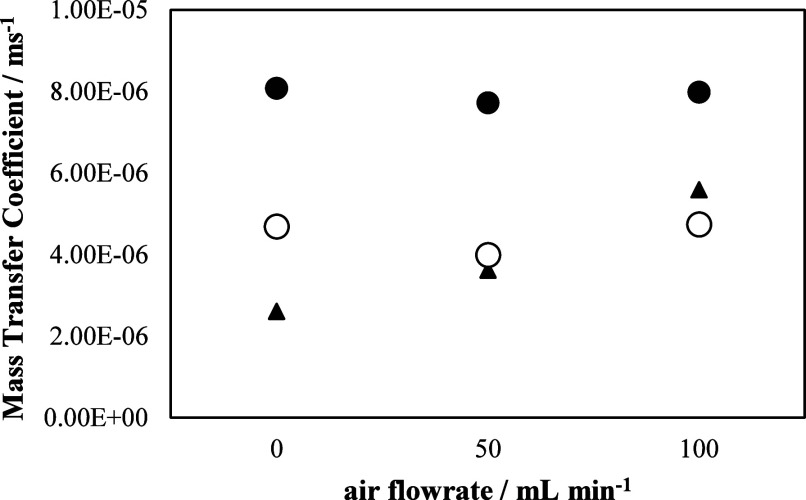
Influence of the gas flow rate on the mass transfer coefficient measured in the cell. ▲Prototype 1; ● Prototype 2 (large electrode size); ◯ Prototype 2 (small electrode size).

As seen, while for Prototype 1, there is a positive influence of the gas feeding through the GDE, Prototype 2 is not influenced by this bubbling. This difference of behavior should be understood in terms of the turbulence produced, and it indicates that as the gas flow rate in the GDE increases, the mass transfer coefficient also increases. This may be due to the very low velocity linear flow in this prototype (5 mm s^–1^), which favors the mass transfer coefficient as the gas flow rate increases, a positive aspect of this mechanical cell design. However, for large Prototype 2, the mass transfer coefficient is barely affected, even when the air flow rate increases. In fact, their values remain very similar across the three experiments conducted. This indicates that the mass transfer coefficient is not being influenced by the gas flow rate entering through the GDE but rather is determined by the system geometry and the velocity of the liquid phase, which is above 50 mm s^–1^. This fact can be explained, in turn, because of the extremely important turbulence associated with the electrolyte flow patterns. Comparing the values obtained in both prototypes, it was observed that values of mass transfer coefficients are much higher in the case of Prototype 2 (by a factor between 1.4 and 3.1 depending on the gas flow fed) and are among the highest reported in the literature for this type of cells.^20^ For the small Prototype 2, we obtain a lower value compared with the large Prototype 2. This could be related to the turbulence and gas flow blocking the small surface area of the electrode, thereby hindering mass transfer and resulting in a lower value. In other words, as with the large Prototype 2, when the gas flow rate increases, the mass transfer coefficient remains constant. However, it is possible that the turbulence generated in this small cell creates an air pocket that blocks the electrode surface, causing a decreasing mass transfer coefficient.

Another important piece of information regarding differences between both cells can be taken from the analysis of the residence time distribution (RTD) curves in the two prototypes. Figure 5 shows those curves obtained with the application of an impulse disturbance using the same feeding flow rate in the three cells and feeding the three different flow rates of gas used in the mass transfer evaluation tests. For these results, curves were determined through impulse response tests, using sodium chloride as a tracer. The tests involved injecting 2 mL of a 5 M sodium chloride solution into the inlet stream and monitoring the outlet conductivity using a Crison GLP 31 conductometer. As seen, there are very important differences in the RTD curves, which supports the important differences observed in the cell performance.

**Figure 5:**
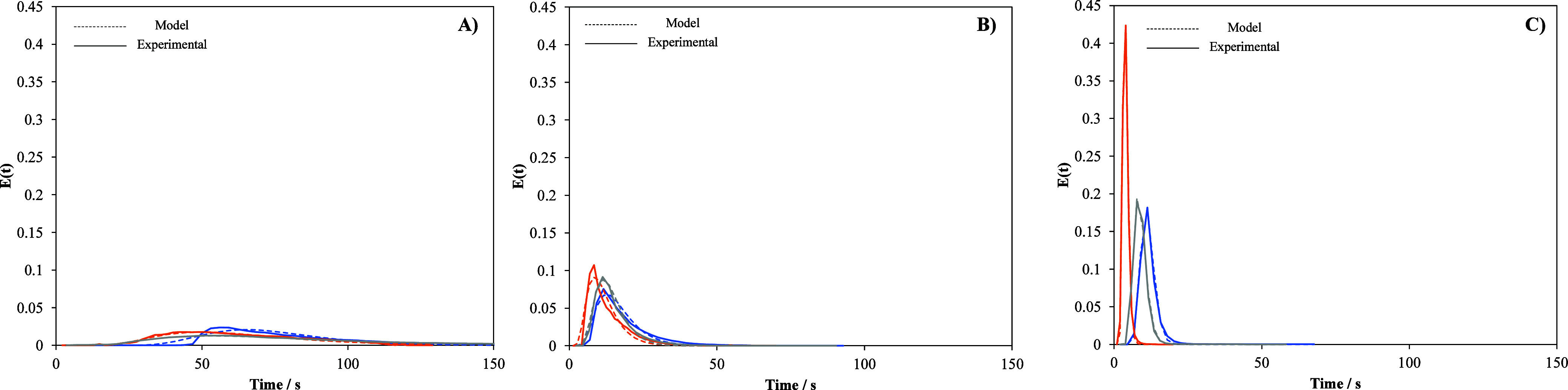
Residence time distribution curves. (A) Prototype 1, (B) Prototype 2 large, and (C) Prototype 2 small. Blue: 0 mL min^–1^; orange: 50 mL min^–1^; gray: 100 mL min^–1^.

To obtain the values of the mean hydraulic residence time (θ) and the reciprocal Péclet number (*D*_L_/vL), experimental data for each cell were fitted to the dispersion RTD model shown in eq 2, where *E*(*t*) stands for the ratio conductivity measured/integral of the conductivity curve with respect to time.

2 $$E\left(t\right)=\frac{1}{2\cdot \theta \cdot \sqrt{\pi \cdot \left(\frac{D_{L}}{vL}\right)\cdot \left(\frac{t}{\theta }\right)}}\cdot e^{-\left(\frac{{\left[1-\frac{t}{\theta }\right]}^{2}}{4\cdot \left(\frac{D_{L}}{vL}\right)\cdot \left(\frac{t}{\theta }\right)}\right)}$$

Values are shown in Figure 6, where the Pe number obtained indicates that in the three cells, the advection is higher than the diffusion transport rates, this fact being more remarkable in the small prototype (Prototype 2). Likewise, the mean residence time indicates a much shorter contact time of the fluid with the electrodes in Prototype 2, which may help to explain differences in terms of the interaction between reaction products generated in the cells, and this is particularly important in the case of H_2_O_2_ electrogeneration as it prevents H_2_O_2_ from self-consuming. In a system where the cathode and anode compartments are separated, there is no scavenger effect, and the H_2_O_2_ concentration is determined by the generation rate and the self-consumption rate of H_2_O_2_ itself. At a certain point, when H_2_O_2_ is in excess, it will naturally tend to self-consume^55,56^

**Figure 6:**
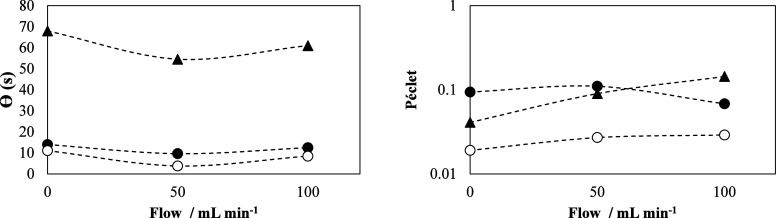
Values of the Pe dimensional number and mean hydraulic residence time obtained by fitting experimental data to model (1) ▲Prototype 1; ● Prototype 2 (large electrode size); ◯ Prototype 2 (small electrode size).

### 3.2 Production of Hydrogen Peroxide

Figure 7 shows the production of H_2_O_2_ using Prototype 1 (conventional flow-by electrochemical cell) in which a linear production of H_2_O_2_ can be seen with time and a positive effect of the increase in the current density on the concentrations reached. Current efficiencies are around 60%, and they do not seem to be affected by the value of the current density, pointing out that further reduction of oxygen to water is not preferred in this device, even by applying harsher conditions. Also, the linear trend observed over the time course indicates the H_2_O_2_ generation rate. This increasing profile is due to the absence of H_2_O_2_ consumption as no scavengers are generated from the anode in this dual-compartment system.^45–49^ It is important to consider that the PEM prevents the crossing of oxidants produced in the anode compartment to the cathode (except for a nondesired crossover), so, initially, only the reduction of H_2_O_2_ on the cathode could account for this competition. Opposite to the negligible effect of the current density on the Faradaic efficiency, an increase in the energy consumption associated with the higher cell voltage produced at higher current densities is observed; that is, the energy efficiency of the process decreases at higher current densities; the average cell voltage values recorded were 2.75, 3.80, and 5.83 V for 25, 50, and 100 mA cm^–2^, respectively.

**Figure 7:**
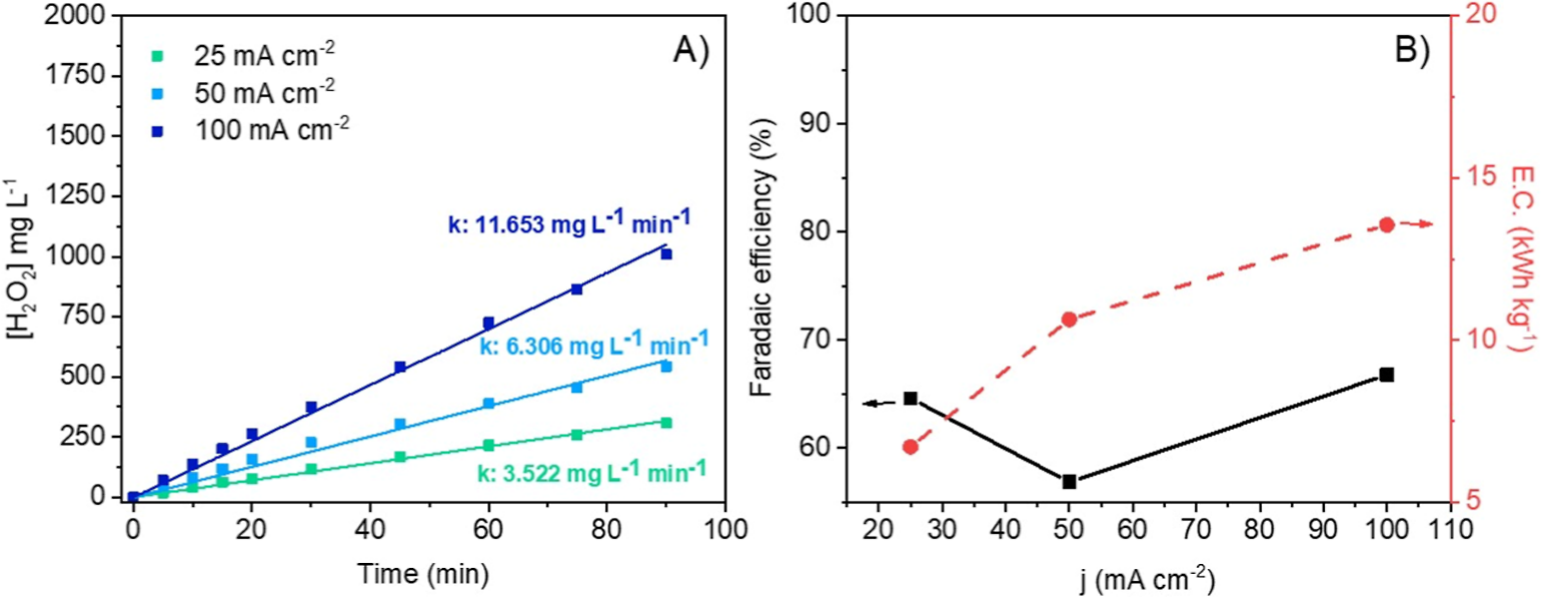
Influence of the current density on the production of H_2_O_2_ with Prototype 1. (a) Time course of H_2_O_2_ production, and k (mg L^–1^ min^–1^) is the constant for H_2_O_2_ electrogeneration. (b) Faradaic efficiency and energy consumption.

Figure 8 shows the results obtained in the same tests when Prototype 1 is replaced with Prototype 2 (with the same electrode area and keeping the same operating conditions). Again, a linear increase of H_2_O_2_ production with time is observed, although in this case, the productions are much higher than those obtained when the Prototype 1 is used because the Faradaic efficiencies increase from the nearness of 65 to more than those of 90%, indicating that, in this case, a positive effect of the increase in current density on the current efficiency can be observed. Faradaic efficiency is directly linked to the applied current and the amount of H_2_O_2_ electrogenerated when an electric current is applied. Comparing both prototypes, distinct Faradaic efficiency values were obtained. To explain this difference, it is important to consider that the same electrodes were used for both prototypes as well as the same applied currents and the gas flow supplied directly to the GDE surface. Therefore, it can be concluded that factors related to the electric current are identical for both prototypes. In this system, the factor influencing Faradaic efficiency must be the amount of H_2_O_2_ electrogenerated in each prototype.

**Figure 8:**
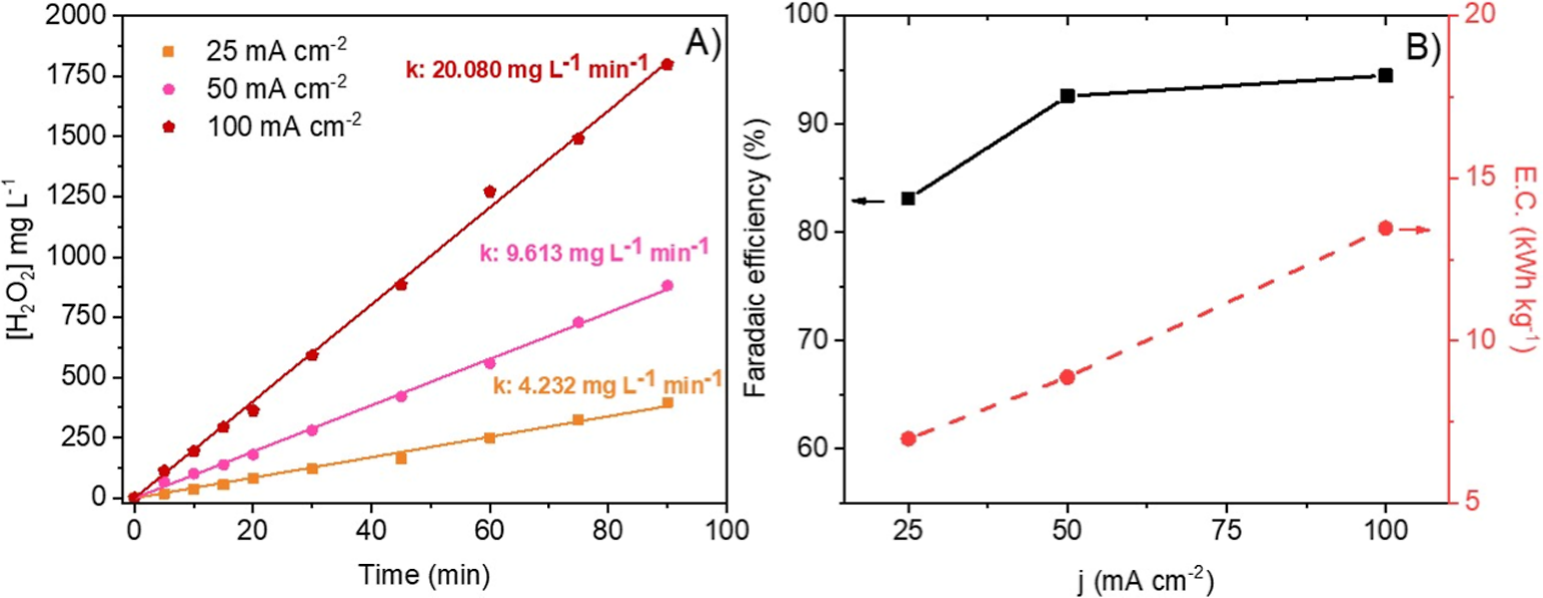
Influence of the current density on the production of H_2_O_2_ with Prototype 1. (a) Time course of H_2_O_2_ production, and k (mg L^–1^ min^–1^) is the constant for H_2_O_2_ electrogeneration. (b) Faradaic efficiency and energy consumption.

H_2_O_2_ electrogeneration is affected by the selectivity of the material (which can be disregarded in this case as the same material was used throughout the study) and by hydrodynamic factors (O_2_ solubility) and mass transfer. Due to mass transport limitations, although convection occurs, diffusion remains the limiting factor for the reaction to proceed. Thus, solubilized O_2_ in the solution must reach the electrode surface to generate H_2_O_2_. Since Prototype 2 exhibits greater hydrodynamics within the reactor, it ensures that the electrogenerated H_2_O_2_ is quickly removed from the electrode surface to the bulk solution. This allows the electrode surface to remain available for the reaction with O_2_. Consequently, Prototype 2 accumulates higher amounts of electrogenerated H_2_O_2_ and demonstrates a higher Faradaic efficiency compared to Prototype 1.

However, this improvement in the current efficiency does not reflect on an important improvement in energy efficiency; because of the larger cell voltages reached in Prototype 2, the average cell voltage values recorded were 6.61, 5.21, and 8.70 V for 25, 50, and 100 mA cm^–2^, respectively. This difference can be attributed to factors such as the gap between the electrodes (cathode and anode), which causes an increase in cell potential, as well as the current collector of each cell. This is worth further evaluation as it highlights another important aspect of electrochemical cell design.

Thus, to go further, voltage–current plots were taken for the three cells. When fitting data to the semiempirical model shown in eq 3, fixing the same values of *a* and *b* for both cells (2.5 and 0.261 V dec^–1^, respectively) because of the use of the same electrodes and reagent feeding, it was obtained that the c parameter doubles from 0.028 to 0.060 ohm cm^2^ when comparing Prototypes 1 and 2 equipped with electrodes of the same size, but in Prototype 2 equipped with the smaller electrodes, a better performance is observed in which c decreases to 0.030 ohm cm^2^.

3 V=a+b·log(j)+c·j

An explanation for this observation can be obtained from the comparison of the current feeders used in both cells, details for which are shown in Figure 9.

**Figure 9:**
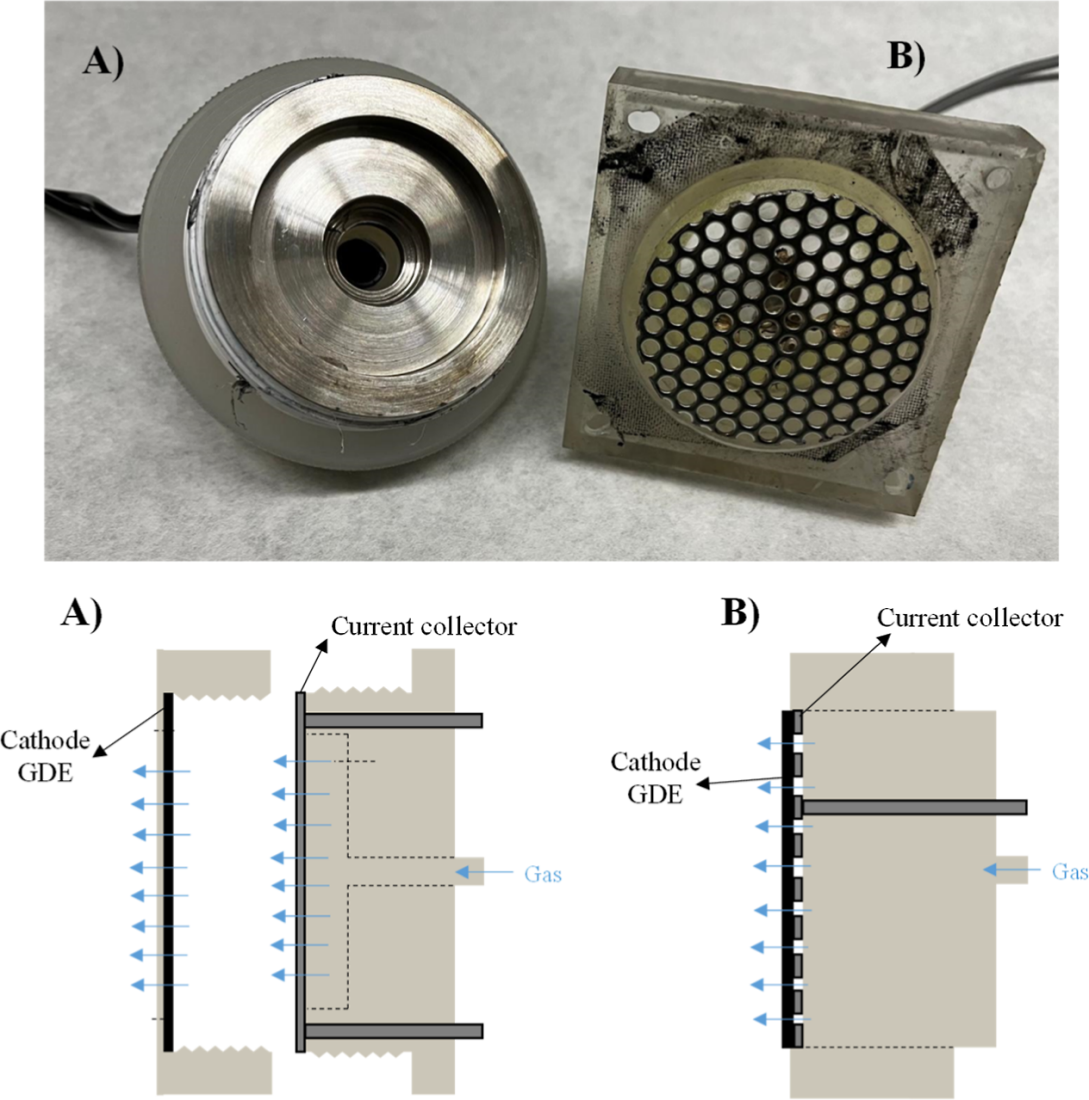
Mechanical details of the current feeders used in Prototypes 1 (part a) and 2 (part b).

Prototype 1 is equipped with a current feeder that undergoes thread tightening, generating very good electric contact. In contrast, Prototype 2 uses a metallic frame with a less intensive contact and that in addition may lose contact in the central positions when gas is flowing because of the deformation produced in the GDE by the pressure, in turn related to the elasticity of the GDE. The extension of the loss of contact is smaller when the electrode is smaller because the ratio between the area of the electrode and the perimeter is more favorable for a good distribution, and this may help to explain the important differences observed when comparing the same size of electrode and how the system improves for the smaller scale, pointing out that mechanical design (not only those related to flows) is very important, especially regarding energy consumption, which directly depends on the cell voltage.

Figure 10 compares the production of H_2_O_2_ for two different sizes of Prototype 2, for which a scale-up factor of 12.5 was applied. For this comparison, scale-up was carefully addressed by maintaining the same specific current density (25, 50, and 100 mA cm^–2^) in both devices. This criterion was achieved by using different electrolyte volumes (1 L for the larger device and 0.080 L for the smaller device) to preserve the scale-up factor while maintaining the same current density. Additionally, the same electrolyte composition (salt and concentration) and gas flow rates were used for both setups.

**Figure 10:**
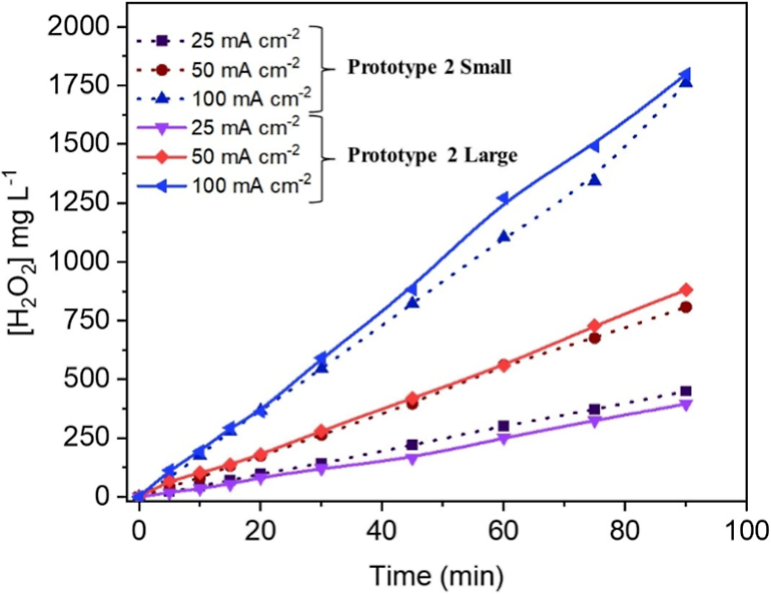
Effect of the scale-up (factor 12.5) in the production of H_2_O_2_ in Prototype 2.

As can be seen, the production of H_2_O_2_ increases linearly in the different tests made, and concentrations reached are very similar, reaching outstanding values, especially considering the large current densities applied. Although differences between the production in both cells seem to be small, it can be seen how the small cell is more efficient for lower current densities; this is consistent with the turbulent flow inside the smaller reactor, which facilitates the evacuation of these gases, and the observation reverses under the more demanding conditions exerted at 100 mA cm^–2^. Initially, this difference was not expected and can be related to the higher production of gases at higher current densities and the difficulties in the evacuation of these gases as well as in the relative volume of gases inside the inner volumes of the cell.

## 4 Conclusions

From this work, the following conclusions can be drawn.

- The mechanical design of electrochemical cells equipped with GDE is a factor of primary importance, and important differences in the fluid dynamic behavior can be obtained with different designs, such as those proposed with Prototypes 1 and 2, despite feeding the same flow rates of electrolyte and gases and using the same electrode composition, area, and interelectrode gap. This fact supports the necessity of more fundamental research on this topic.
- Centrifugal flow patterns as those obtained using Prototype 2 are very effective in promoting electrochemical reactions in cells equipped with GDE, and this can be explained in terms of the high turbulence and efficient release of gases that help attain them. With these patterns, mass transport coefficients do not significantly depend on the flow rate of gases fed, opposite to cells with parallel flow patterns in which the feeding of gases may even double the value of the mass transport coefficients. In equivalent conditions, mass transport coefficients are between 1.5 and 3 times higher in Prototype 2 than in Prototype 1.
- By using single flow cells with flow patterns parallel to the electrodes, such as those produced in Prototype 1, Faradaic efficiencies as high as 66.7% in the production of H_2_O_2_ and energy consumptions of 13.5 kW h kg^–1^ can be obtained.
- By using tailored centrifugal flow patterns, such as those obtained in Prototype 2, Faradaic efficiencies higher near 90% and energy consumptions of 13.4 kW h kg^–1^ can be attained.
- Scaling up by increasing size of electrodes shows no influence on the values reached in the production of H_2_O_2_. In contrast, the less efficient cell voltage attained suggests the necessity of considering other factors, which typically are less studied in scientific literature such as the design of the current feeders. This has an important impact on energy efficiency.

## Supplementary Material



## Notes

Polarization curves (I vs V) by the current limiting method for the different prototypes and flowrate gas: 0 ml min^‑1^, 50 ml min^‑1^, and 100 ml min^‑1^ (PDF)
