# Supplementary material for: Boosting New Electrochemical Reactor Designs to Improve the Performance in H2O2 Production Using Gas Diffusion Electrodes
Source: ACS Sustain Chem Eng. 2025 Feb 20;13(8):3172–82. doi: 10.1021/acssuschemeng.4c08826 (PMC12124220; doi:10.1021/acssuschemeng.4c08826)
Supplement: Supplementary file 1 [file sc4c08826_si_001.pdf]

Supporting Information

Boosting new electrochemical reactor designs to improve the performance in the H_2O_2 production using Gas Difussion Electrodes

Taynara Oliveira Silva[1,2,+], Rafael Granados-Fernández[2,+], Justo Lobato[2], Marcos R V Lanza[1*], Manuel Andrés Rodrigo[2,*]

1 São Carlos Institute of Chemistry, University of São Paulo (USP), 400, 13566-590, São Carlos, SP, Brazil

2 Department of Chemical Engineering, Universidad de Castilla-La Mancha,13071 Ciudad Real, Spain

Table of Contents

1. **Polarization curves (I vs V) – Current limiting method…………………...S1**

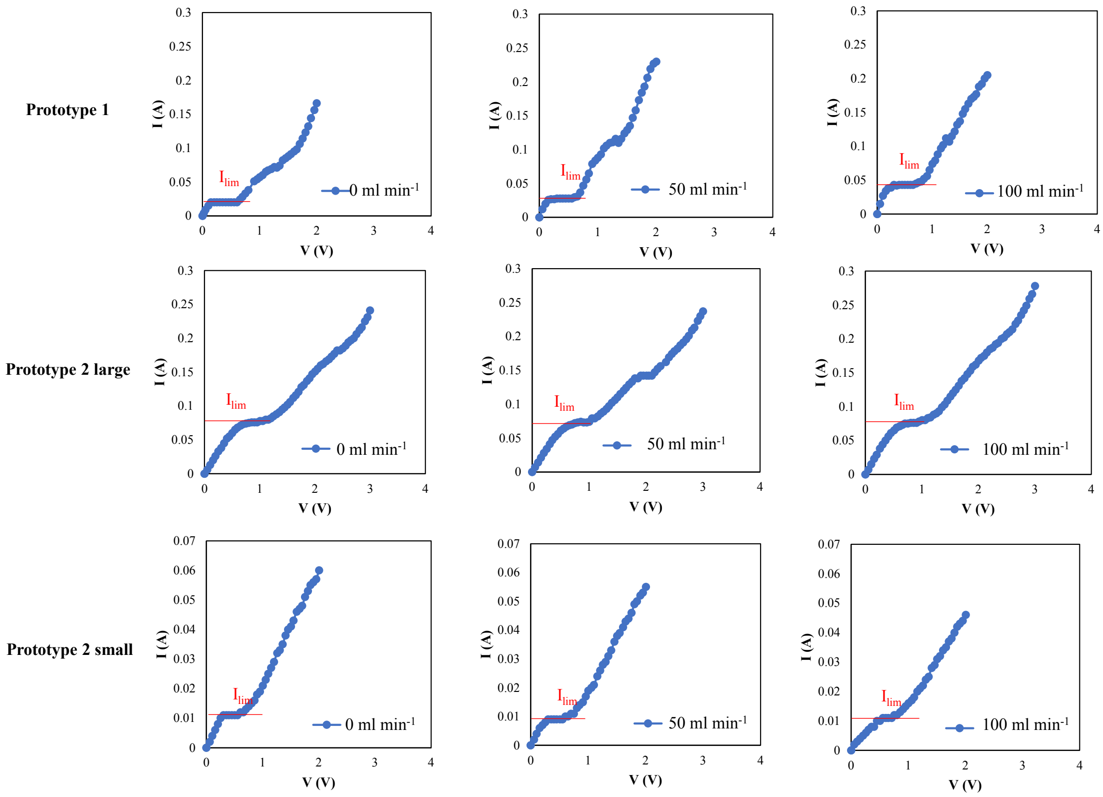

Figure S1. Polarization curve (I vs V) by current limiting method for the different prototypes and flowrate gas; 0 ml min^{-1}, 50 ml min^{-1}, and 100 ml min^{-1}.
